# P03 Comparison of direct and standard sensitivities of Enterobacterales in blood cultures

**DOI:** 10.1093/jacamr/dlaf230.010

**Published:** 2025-12-04

**Authors:** Bushra Sultan, Andrew McArdle, Mukul Acharya

**Affiliations:** Alder Hey Children's Hospital NHS Foundation Trust, Liverpool, UK; Alder Hey Children's Hospital NHS Foundation Trust, Liverpool, UK; Alder Hey Children's Hospital NHS Foundation Trust, Liverpool, UK

## Abstract

**Background:**

Rapidity and reliability of antimicrobial susceptibility testing is particularly important for bloodstream infections. Studies have shown that mortality from sepsis increases every hour that the time to first appropriate antimicrobial is delayed. Direct antimicrobial susceptibility testing (DAST) applied on samples directly from positive blood culture bottles for faster antibiotic sensitivities potentially leading to the appropriate treatment for bloodstream infections.

**Objectives:**

To compare the direct and standard blood culture sensitivities to ascertain the reliability of the antibiotics for the Enterobacterales. The blood cultures with Gram-negative bacilli on microscopy were worked up for DAST, ID and standard AST subsequently and compared.

**Methods:**

All the blood cultures growing Enterobacterales in the time period of 24 months specifically from years 2023 and 2024 were included. The data were analysed using Microsoft Excel sheets and 2×2 pivot tables were used to calculate the sensitivity, specificity, positive predictive values and negative predictive values.

**Results:**

The direct disc diffusion antimicrobial susceptibility for Enterobacterales in blood cultures is fairly reliable with sensitivities for co-amoxiclav, ciprofloxacin and piperacillin/tazobactam (98.14%, 93.75% and 90.90%, respectively) and less reliable for gentamicin (72.2%). The specificities are above 90% for meropenem, ciprofloxacin, gentamicin and piperacillin/tazobactam (98.90%, 96.82%, 96.15%and 91.89%, respectively)

**Conclusions:**

We recommend review of the sensitivities and specificities with a larger data for example over a 3 years’ time for better representation of the Enterobacterales susceptibilities data of the direct disc diffusion test of the antibiotics as compared to the standard susceptibilities due to increasing resistance.

